# The *Cannabis sativa* genetics and therapeutics relationship network: automatically associating cannabis-related genes to therapeutic properties through chemicals from cannabis literature

**DOI:** 10.1186/s42238-023-00182-z

**Published:** 2023-05-30

**Authors:** Trever J. Jackson, Sunandan Chakraborty

**Affiliations:** 1 M.S. in Bioinformatics, Luddy School of Informatics, Computing, and Engineering, Indiana University IUPUI, Indianapolis, IN USA; 2 Human-Centered Computing, Luddy School of Informatics, Computing, and Engineering, Indiana University IUPUI, Indianapolis, IN USA

**Keywords:** *Cannabis sativa*; Natural language processing; Relationship extraction; Knowledge graph; Cannabinoids; Terpenoids; Natural medicine; Plant genetics

## Abstract

**Background:**

Understanding the genome of *Cannabis sativa* holds significant scientific value due to the multi-faceted therapeutic nature of the plant. Links from cannabis gene to therapeutic property are important to establish gene targets for the optimization of specific therapeutic properties through selective breeding of cannabis strains. Our work establishes a resource for quickly obtaining a complete set of therapeutic properties and genes associated with any known cannabis chemical constituent, as well as relevant literature.

**Methods:**

State-of-the-art natural language processing (NLP) was used to automatically extract information from many cannabis-related publications, thus producing an undirected multipartite weighted-edge paragraph co-occurrence relationship network composed of two relationship types, gene-chemical and chemical property. We also developed an interactive application to visualize sub-graphs of manageable size.

**Results:**

Two hundred thirty-four cannabis constituent chemicals, 352 therapeutic properties, and 124 genes from the *Cannabis sativa* genome form a multipartite network graph which transforms 29,817 cannabis-related research documents from PubMed Central into an easy to visualize and explore network format.

**Conclusion:**

Use of our network replaces time-consuming and labor intensive manual extraction of information from the large amount of available cannabis literature. This streamlined information retrieval process will enhance the activities of cannabis breeders, cannabis researchers, organic biochemists, pharmaceutical researchers and scientists in many other disciplines.

**Supplementary Information:**

The online version contains supplementary material available at 10.1186/s42238-023-00182-z.

## Background

Thousands of years in the past, the ancestors of modern man foraged the prehistoric landscape of Central Asia for botanical sources of relief from common injuries and ailments such as broken bones and gum disease. Plant species with medicinal use dating to this period include *Cannabis sativa* (hemp and marijuana), *Piper betel* (betel nut), and *Papaver somniferum* (opium poppy) (Hardy 2021). This ancient behavior has evolved into the modern pharmaceutical industry, where many drugs, including most anesthetics and analgesics, are derived from plant compounds. For ex ample, the “prototype of modern local anesthetics” is cocaine from the *Erythroxylum coca* species (Tsuchiya 2017).

*Cannabis sativa* evokes it is medicinal power from an array of secondary metabolites encompassing several chemical classes, including terpenoids and flavonoids. Cannabis is the sole botanical producer of cannabinoids, the class of terpenoids which interact with the mammalian endocannabinoid receptors. This fact has led to much effort in examining all cannabis chemicals, notably the cannabinoids delta-9-tetrahydrocannabinol (THC) and cannabidiol (CBD), for therapeutic effects such as analgesia, psychoactivity, and antiviral (Raj et al. 2021; Linher-Melville et al. 2020; Jagannathan 2020).

Cannabis chemicals are routinely investigated individually, as well as in combination, to explore entourage effect (Raj et al. 2021; Linher-Melville et al. 2020; Jagannathan 2020).

**Fig. 1:**
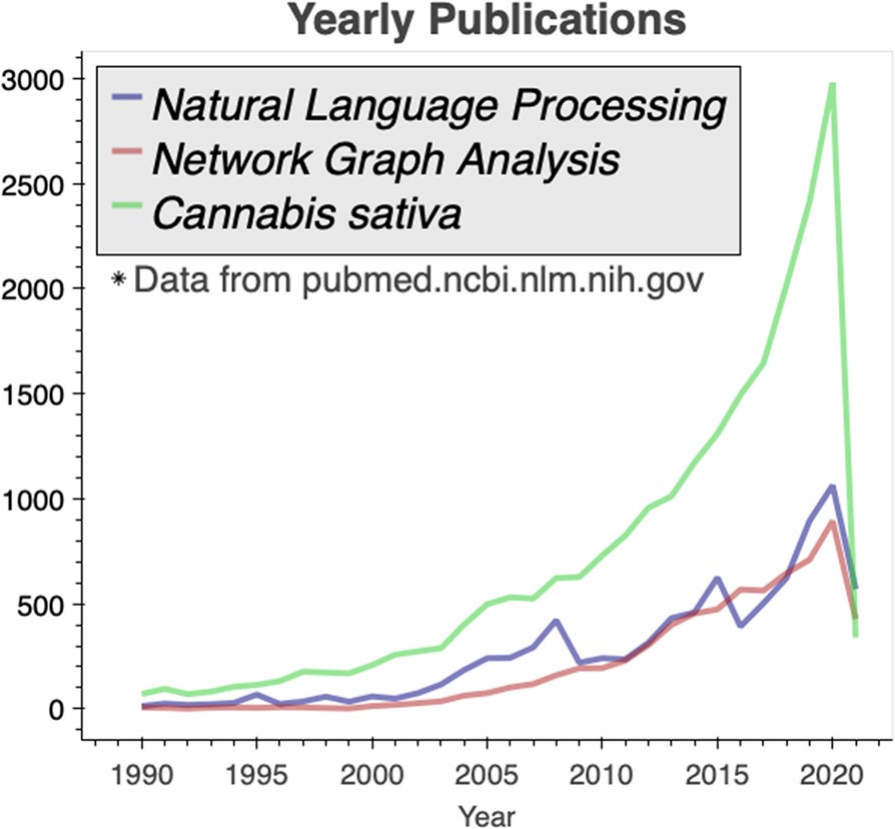
Concurrent increase in publications related to cannabis, natural language processing, and network graph analysis. Data from PubMed in May of 2021

The study of the genetic basis for the chemical composition of cannabis began in earnest only after the publication of a reference genome and transcriptome in 2011 for the Purple Kush strain (van bakel 2011). PubMed has since exploded with cannabis-related citations (Fig. 1 green line). The cannabis genome assembly has been rapidly refined, and many transcriptomic studies have been performed and published. Sen Cai et al. have recently expertly assimilated the available cannabis bioinformatics sequencing data and strain-specific metabolite analyses into a useful resource for cannabis researchers (Cai 2021).

However, the cannabis domain still lacks a resource to access detailed information about the therapeutic nature of each cannabis chemical constituent alongside relevant genetic information. Our work fills this gap by using automatic relationship extraction methods from 29,817 cannabis-related biomedical documents from PubMed Central (cannabis corpus) to provide a map of genetics to therapeutic effects induced by plant chemicals. This “database” of the gene-to-chemical and chemical-to-property relationships allows for instant retrieval of a complete property and gene set for any chemical appearing in the cannabis corpus. Knowledge of plant genes to therapeutic property connections is essential to inform the future selective breeding of cannabis strains (van bakel 2011). We capture this information and present it within an intuitive network structure for the end-user to explore relationships and retrieve information.

The concurrent upward trend in yearly publications for three scientific areas relevant to our work is shown in Fig. 1. Natural language processing (NLP) methods for performing automated information extraction from text or speech have become increasingly important to combat the “information overload problem” experienced in the biomedical domain (Neumann et al. 2019). Information overload is also commonly tackled through the use of network graph structure. Many scientific problems inherently fit network structure and analysis. Because of this, network graph machine learning strategies and graph convolutional networks have received particular research attention of late (Leskovec 2021). We combine these burgeoning areas to drive cannabis research forward by providing access to information that is otherwise costly to procure.

Manually producing a list of all genes or therapeutic properties associated with even one cannabis chemical requires a complete scan for relevant scientific literature and labor-intensive discovery and documentation of relationships. We, therefore, developed an NLP pipeline to automate these tasks and applied the pipeline to a large amount of available cannabis literature to produce a unique multipartite relationship network of genes, chemicals, and properties, with chemicals playing the central role in linkage. Besides establishing which relationships exist, the network stores information about the literature sources for each relationship, allowing the accessible location of documents regarding a topic. The network is helpful for many scientific research areas and can save valuable time and labor when researching cannabis-related chemicals, genes, or therapeutic properties.

## Methods

### Data collection

We developed a web scraping pipeline to collect *Cannabis sativa-*related research articles from PubMed and PubMed Central (PMC). These repositories provide access to citation records for scientific publications. These records include a publicly accessible link to full text for open-access documents. PubMed was chosen as a starting point for literature resources due to its well established reputation, partly owing to its relationship with the NIH in which all NIH funded data must make findings available to the public via PMC. PMC publications are generally therefore open-access which allows for automated webscraping. PubMed also comes with the methodological advantage of Biopython’s bio.entrez package which allows python-based programmatic access to the NCBI Entrez API to search all NCBI-related databases for information, in our case, cannabis-related publications from PMC. The inclusion of non-overlapping documents from other literature resources would prevent the inherent bias in utilizing only PMC publications. Other sources of documents that could be mined for information are Google Scholar, ScienceDirect, and Europe PMC, Europe PMC already coordinates data with PMC however and the increased methodological complexity as well as increased data size associated with including documents from Google Scholar and ScienceDirect present hurdles to be overcome in future updates of the network.

We utilized bio.entrez to obtain a list of document identification numbers returned for the search term ‘Cannabis sativa’. The list of 30,000 document IDs dated from January 1853 to January 2021 and the Bio.Entrez.efetch() function enabled us to remotely access an xml file for each document at which time the python package Beautiful Soup 4 (bs4) was used to scrape relevant text information from the xml code. The built-in bio.entrez and bs4 functions appropriately handle requests for information so as not to overload the host servers. Many non-English language documents were observed; therefore, another python package, TextBlob, was used to scan titles and omit non-English documents. This resulted in a final text dataset forged from 29,817 cannabis research articles collected from the PubMed Central database. We also collected the article abstracts and meta-data, such as title, author names, journal names, publication date, and the doi link for included documents. We call the dataset the *Cannabis Corpus*. The data set has been made available along with python code and extended methods through a publicly accessible link.^1^

### Extraction of gene, chemical and therapeutic properties

Some NLP tasks, such as part-of-speech tagging, are ubiquitous to the field and therefore thoroughly explored. More specific tasks such as gene name recognition and chemical name recognition are commonly of interest to biologists leading to public availability of pre-trained models for these tasks such as those supplied by Scispacy. Even so, intricate aspects of the human language are difficult to model even if the basic principles involved are simple. Adjectives may be easy to predict, as Scispacy comes loaded with an automated part-of-speech tagger. However, our specific problem of accounting for only adjectives that are biomedical or therapeutic descriptors of chemical compounds is complex enough that no existing method is readily available to automatically accomplish this task. We therefore compiled a seed set of preliminary therapeutic properties (properties) by manual curation from a subset of cannabis and essential oil-related documents deemed to be most useful for this purpose (Tsuchiya 2017; Jagannathan 2020; Zager 2019). Then to augment the capabilities of humans we extended this list by searching for semantically similar words and phrases using word embedding in the cannabis corpus documents. Word embedding is a real-valued vector representation of words for text analysis that encodes the word’s meaning such that the words closer in the vector space are expected to be similar in meaning. We created word vectors from the cannabis corpus with the python package Gensim using word2vec skip-gram model (Řehůřek, R., Sojka, P. 2010). We rapidly extracted new properties by searching for words within a small radius in the word vector space from the manually curated list. We compiled a comprehensive list of 702 phrases representing therapeutic concepts. We used cosine similarity (Schu¨tze, H., Manning, C.D., Raghavan, P. 2008) to compute the distance between two words in the word2vec vector space.

We used a pre-trained model to extract other relevant biomedical entities from the text during processing. We aimed to label each word in the sentences extracted from the cannabis corpus articles. This helped identify genes, chemical, and therapeutic properties mentioned in the articles, and their inter-relationships.

We used four labels to represent the above three entities and an additional label-*None* to represent words that do not belong to the above three categories. ScispaCy provides state-of-the-art pre-trained models for part of speech tagging, sentence segmentation, and biological named-entity recognition (bioNER) (Neumann et al. 2019). During relationship extraction, genes and chemicals were predicted with pre-trained ScispaCy bioNER models, while therapeutic properties were located by cross-referencing paragraph words with the properties list. The use of ScispaCy to locate gene names and chemical names alleviated the need for the time-consuming production of custom bioNER models.

### Relationship extraction

Many past studies have conducted research on cannabis-related genes, and some of them are represented in the cannabis corpus. The findings of all these researches are distributed across thousands of published articles. Often these research articles also present findings about how two or more genes are related or have similar or related roles. These relationships can provide valuable insights, but there is no existing knowledge base that records conglomerate information for such relationships as they pertain to therapeutic properties. Whereas manually browsing all research articles and curating this information is infeasible, we used the terminology extraction method discussed above to efficiently encapsulate information on relationships between cannabis genes, chemicals, and therapeutic properties in a convenient network format.

Briefly, the natural structure of language into paragraphs of related content lead us to process the data on a paragraph by paragraph basis. We identified gene and chemical names from each paragraph of the fully concatenated cannabis corpus articles using the pre-trained SciSpacy models (Daiber et al. 2013; Rodriguez-Esteban 2009; Zhou et al. 2014; Finkel et al. 2005). Given a gene *g* and mentioned in paragraph *d* for chemical (Φ_*d*_) found in *d*. The relationship between *g* and chemical *a* (s.t. *a* ∈ Φ_*d*_) was determined by the likelihood of them co-occurring compared to them occurring independently. Given two entities *g* and *a*, if *p*(*g*) and *p*(*a*) represent the probability of *g* and *a* in the data (i.e., the cannabis corpus), then the relationships *p*(*a*) ≈ *p*(*a, g*) “and”/ “or” *p*(*g*) ≈ *p*(*a, g*) denotes a connection between *g* and *a*. Here, “and” represents a bi-directional relationship, i.e., both the relationships *p*(*a*) ≈ *p*(*a, g*) *and p*(*g*) ≈ *p*(*a, g*) are true. On the other “or” denotes a unidirectional relationship, i.e., either *p*(*a*) ≈ *p*(*a, g*) is true *or p*(*g*) ≈ *p*(*a, g*) is true. If only *p*(*a*) ≈ *p*(*a, g*) is true, it will denote the unidirectional relationship *a* → *g*. To validate this (almost) equality, we used the likelihood ratio hypothesis test (Manning and Schutze 1999).

We also located therapeutic properties in each paragraph and extended this co-occurrence-based method to capture relationships from chemicals to properties. Each paragraph of the corpus was scanned for gene, chemical, and property entities; thereafter, edges were added to the network graph for each pair of entities of gene-chemical and chemical-property type. Additional edge weight is determined by the product of the number of in-paragraph occurrences for each entity. The network also includes an entity type of “strain,” and edges from strain to each chemical constituent reported in a study of 9 cannabis strains by Zager et al. (2019) was manually added to examine groups of chemicals according to strain.

### Implementation

All programming was performed with Python 3.7.4 with the following package versions: Beautiful Soup 4.8.0, ScispaCy, Networkx 2.3, Bokeh 1.3.4, PubchemPy 1.0.4, Gensim 3.8.0, BioPython 1.76 (Neumann et al. 2019; Richardson 2007; Hagberg et al. 2008; Bokeh Developement Team 2018). The custom-made bokeh application for information query, network exploration, and visualization will be made available to the public as a website and as code to re-build the network from scratch on github. We intend to update the network to include data from newly published PubMed Central Open Access cannabis-related documents at 6-month or annual intervals.

## Results

### Network structure

The *Cannabis sativa* Genetics and Therapeutics Network consists of 29,862 vertices or nodes (20,495 genes, 8,978 chemical, 380 properties, and 9 strains) connected by 389,334 links or edges. Each node is assigned a degree attribute defined as the number of edges in which the node participates. The average node degree for our network is 26. Node degree also signifies the number of direct neighbors possessed by a node. Large networks containing nodes with many neighbors can be challenging to visualize. We, therefore, developed an interactive dashboard to query any node or set of nodes from the network and project those nodes and neighbors into a sub-graph that is human readable.

### Example gene to therapeutic property linkage

**Fig. 2:**
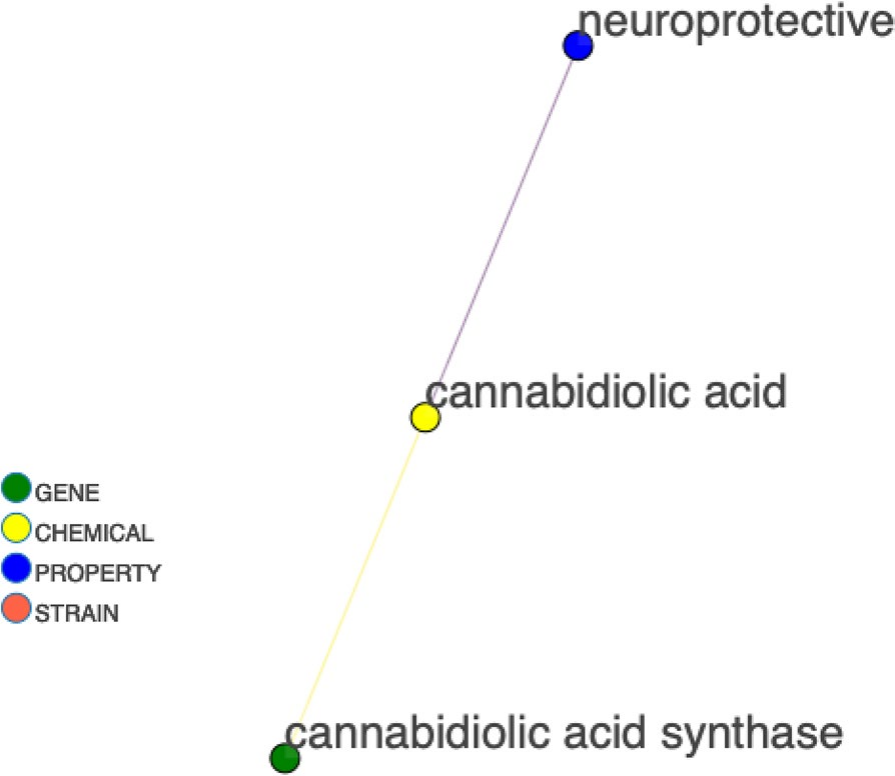
Synthase gene to therapeutic property linkage. Sub-graph for CBDA synthase and neighbors

Figure 2 shows the sub-graph for cannabidiolic acid synthase (CBDAS), the gene that synthesizes cannabidiolic acid (CBDA), the form of CBD stored in plants. The neighbors of the gene include chemical precursors, such as cannabigerolic acid (CBGA) and olivetolic acid, as well as the end product CBDA. A limitation of our current approach is the lack of specific labels for relationships. However while processing the paragraph data, the paragraphs responsible for each relationship are stored as an edge attribute with that edge. This text information is recallable by the end-user. Currently in the interactive network version, the end-user can click on an edge to populate a window with supporting paragraphs to obtain information on the relationship of the connected entities. In the future, this text data can be processed for each edge to provide an automatically extracted label for the relationship type, such as “precursor” or “product”.

**Fig. 3:**
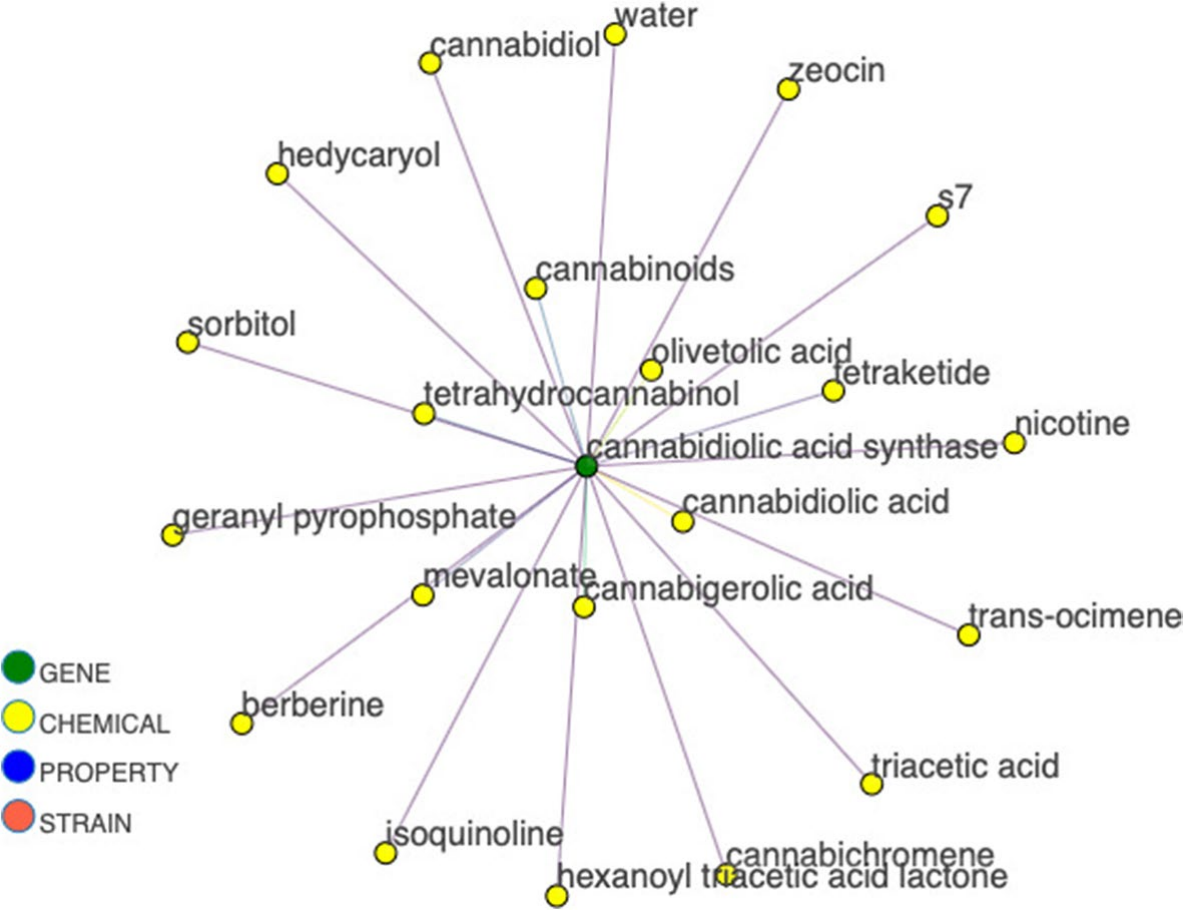
Synthase gene to therapeutic property linkage. Example of gene to property linkage for CBDA synthase

CBDA, although most often converted by heat to CBD before consumption, is also therapeutically active (Pellati 2018). CBDA links to several properties other than neuroprotective, and it can be assumed that varying action of CBDAS would modulate these therapeutic effects through CBDA concentration. The gene responsible for CBDA production (CBDAS) can then be observed in relation to the properties of CBDA. Figure 3 displays this relationship as a simple example of gene linkage to property through a chemical. This same analysis can be carried out for any cannabis gene.

**Fig. 4:**
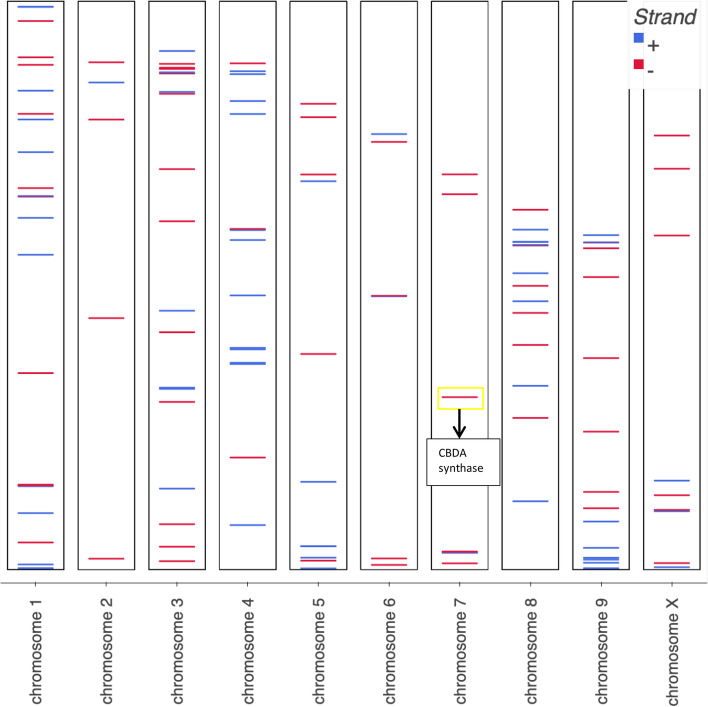
Chromosomal distribution of cannabis genes with therapeutic property linkage. CBDA synthase is located on chromosome 7. Chromosomes not shown true relative size

Our approach identified 124 genes from a list of genes obtained from the annotation of the *Cannabis sativa* reference genome. These genes possess links to a set of properties through a set of chemicals. These sets of genes, chemicals, and properties and the relationships between them represent a bountiful research opportunity. The chromosomal distribution of these genes in the cannabis genome is shown in Fig. 4 and the CB-DAS is highlighted on chromosome 7.

### Reporting therapeutic properties associated with monoterpenes

**Fig. 5:**
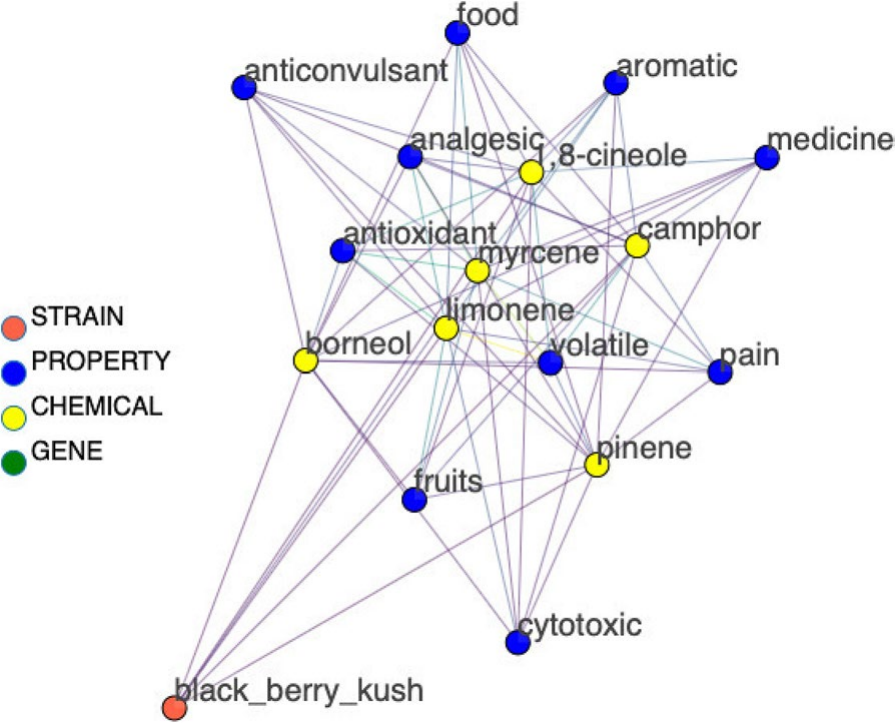
Sub-graph showing therapeutic properties associated with a group of monoterpenes commonly found in Blackberry Kush

Retrieving a complete set of therapeutic properties for a chemical or set of chemicals is a strength of the network. Figure 5 shows the sub-graph for a set of six monoterpenes commonly observed in Blackberry Kush (Zager 2019). The sub-graph is filtered to show only nodes of degree 6 and higher.

**Fig. 6:**
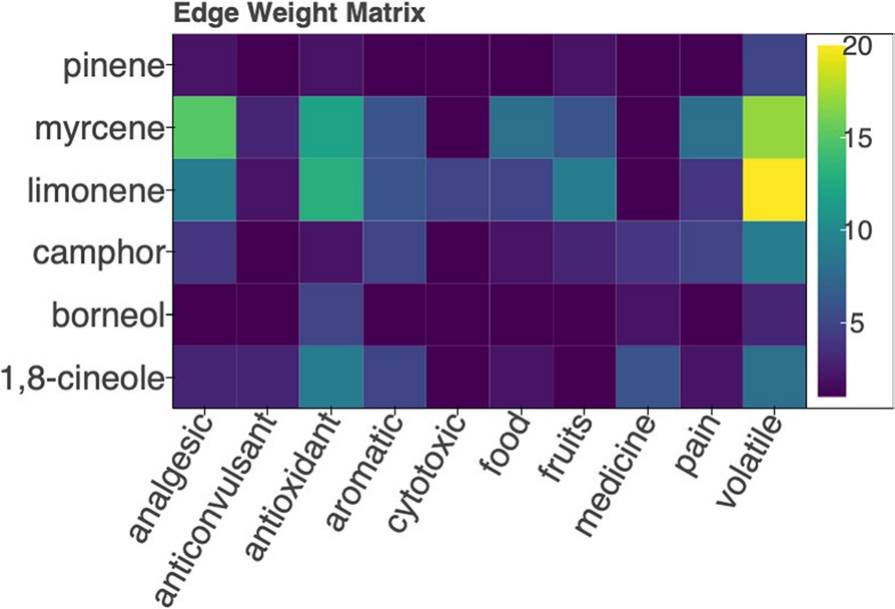
Heatmap visualization of node degrees for nodes in Fig. 5

Each edge in the network is assigned a weight attribute in addition to supporting paragraph and citation attributes. The edge weights for a sub-graph can be visualized as a heatmap to aid visual analysis. Detailed methods for assigning edge weight are provided in the supplemental material (see Section A3). Figure 6 shows a heatmap for the sub-graph in Fig. 5. The brightly colored areas represent the highest weighted links, or more commonly co-occurring entities, limonene, and myrcene, in relation to volatile, antioxidant, and analgesic properties. Therefore, a cannabis strain with high quantities of monoterpenes may likely impart these therapeutic effects. Dark areas of the heatmap represent low-weighted relationships. These relationships could be either therapeutic dead ends or uncharted research territory. In this manner, researchers can analyze groups of chemicals and determine likely contribution to therapeutic effect, and identify novel areas for investigation.

### Investigating genes underlying CBD-antiviral relationship

**Fig. 7:**
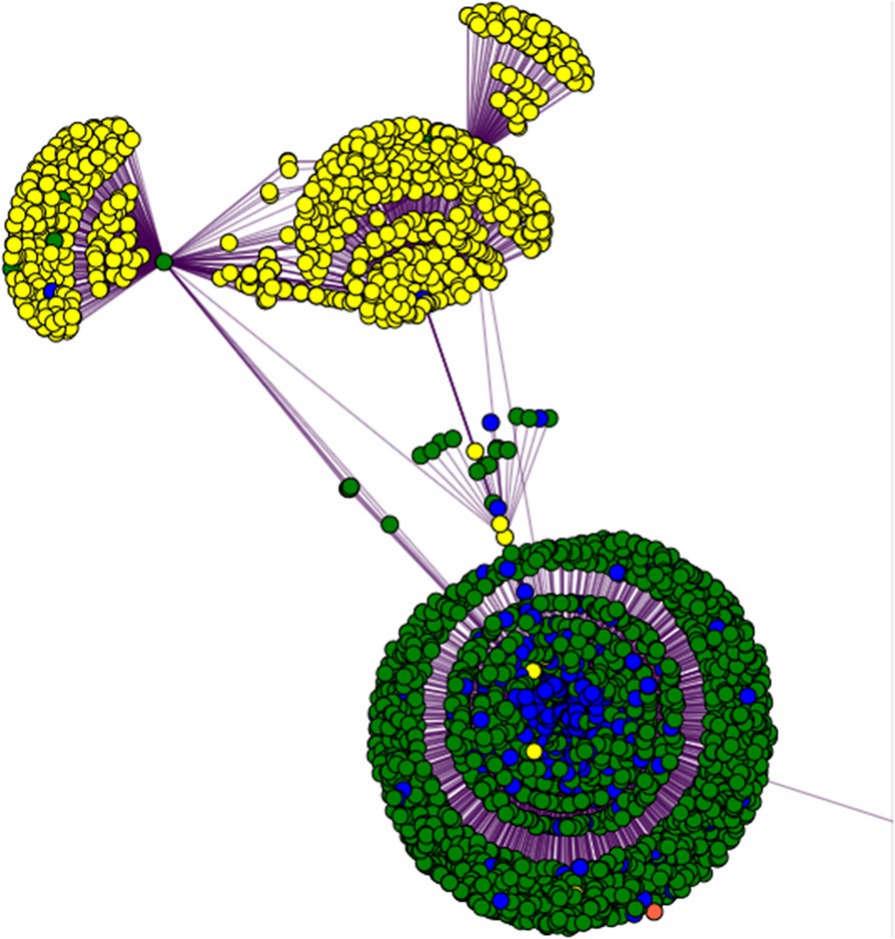
The beginning stage of cross-referencing gene connections for two chemicals, CBD and ribavirin, known to be related to antiviral property. Gene nodes are colored green, chemical nodes are yellow, property nodes are blue, and strain nodes are orange. The high node degree of these nodes make human interpretation difficult at this point, therefore filtering of nodes is necessary. We can filter out nodes with low degree to make the more highly connected components more visible

**Fig. 8:**
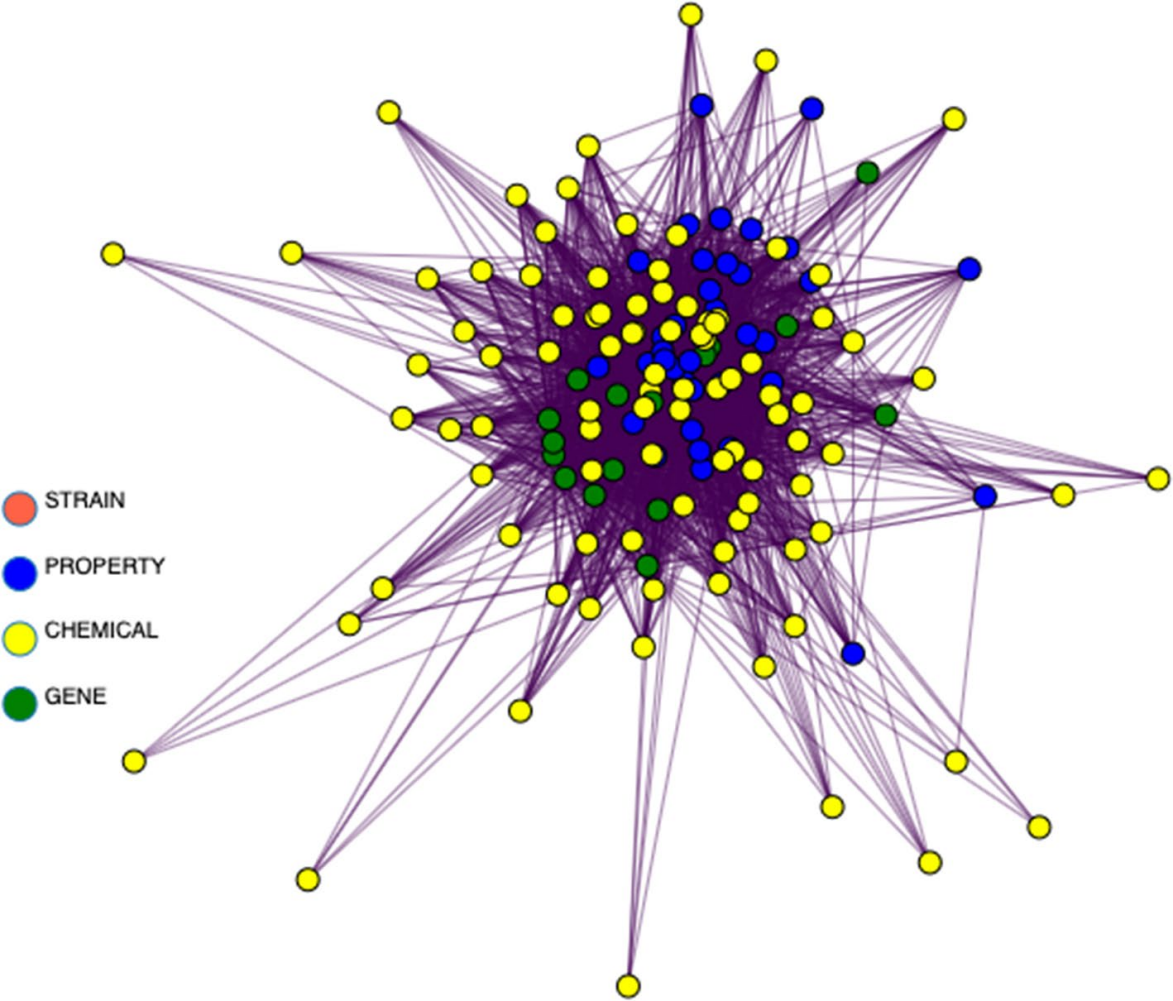
Figure 7 was filtered to exclude nodes with degree less than 4 to produce this condensed version of the graph. We can further filter by node degree less than 5 to produce Fig. 9

**Fig. 9:**
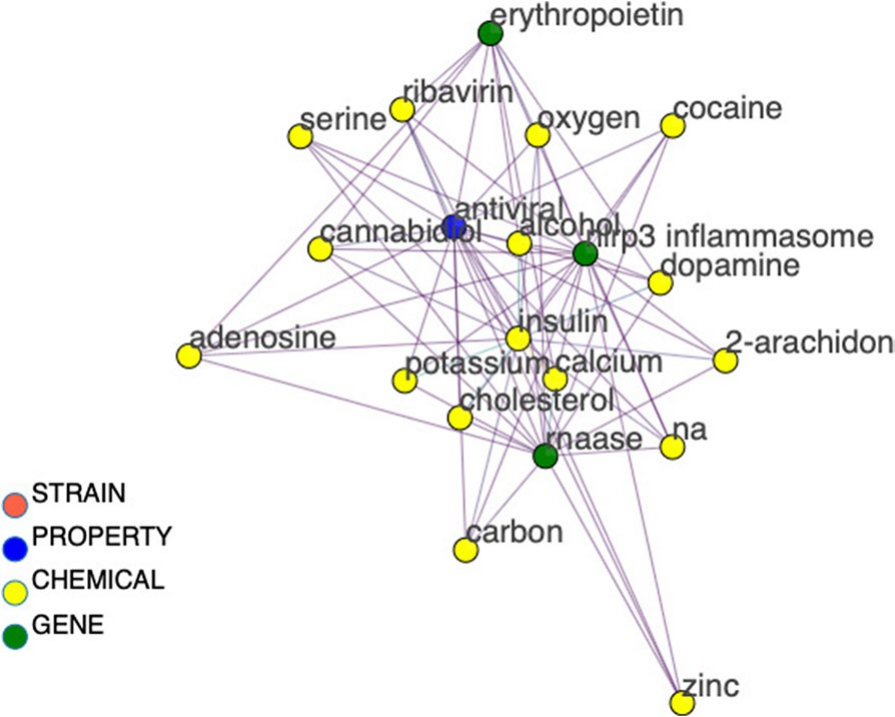
The graph is now condensed enough to be interpreted by a human. Node labels are added, via a toggle switch on the interactive bokeh visualization. A final node degree filter is applied to filter out nodes with degree less than 9 to produce Fig. 10

**Fig. 10:**
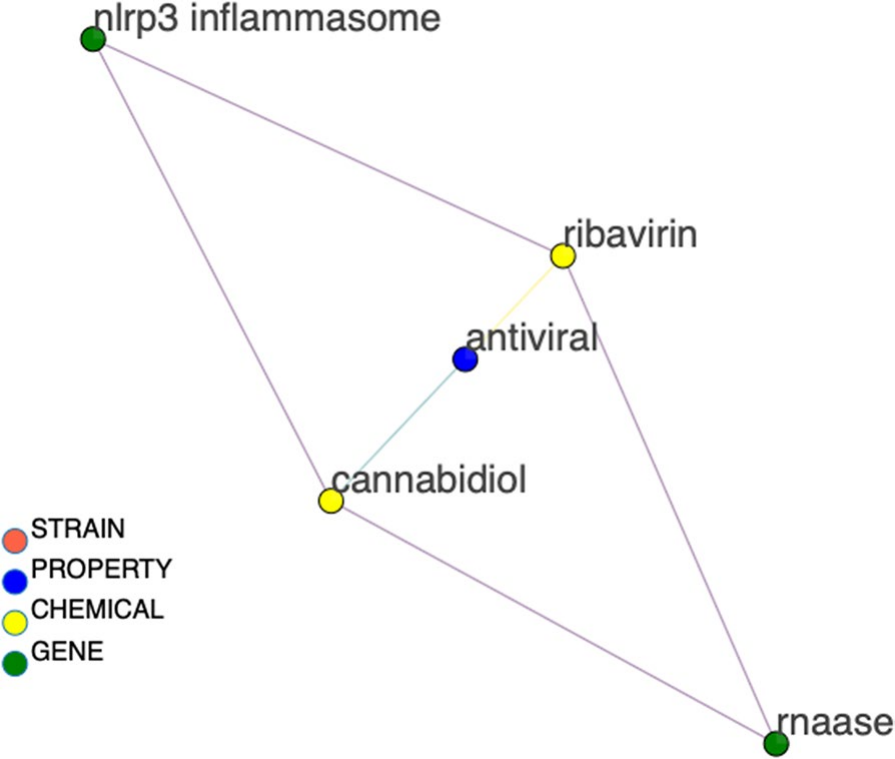
Final condensed version of the CBD, ribavirin, and antiviral cross-reference sub-graph clearly showing the requisitioned common gene connections

Figures 7, 8, 9, and 10 depict the progressive filtering of a subgraph by node degree for a query of “ribivarin”,”CBD”, and”antiviral” from the bokeh network visualization tool. The filtering can be per-formed on a slider after query in the interactive network version and brings about better visualization of the targeted relationships. This example query represents a research strategy of cross-referencing known chemical neighbors of a property to elucidate common gene relationships. We cross-reference gene neighbors of known antiviral chemical, ribavirin, with those of CBD to assess mutual gene connections of the chemicals. The initial graph is cluttered however once the graph is progressively filtered, it is easy to see that”rnaase”, and”nlrp3 inflammasome” are shared gene neighbors of”CBD”, “ribavirin”, and”antiviral”, implying these genes may play a role in the antiviral activity of these chemicals. Note that the local node degree shrinks for each node as the local graph is filtered and nodes are removed.”Antiviral” was initially highly weighted with many connections. The edge between “CBD” and “nlrp3 inflammasome” has a weight equal to one with the source document reporting “flavonoids with inhibitory effects on acute lung injury” and “the mechanisms underlying the inhibitory effects of natural terpenoid compounds on ALI (acute lung injury)” (He 2021). This information could be useful for drug discovery targeting viral lung disease, such as COVID-19. This example demonstrates how the network could be a valuable resource to mine for potential natural treatments of many diseases.

## Discussion

The network is a first-of-its-kind knowledge base for *Cannabis sativa* and functions as a time-saving resource for retrieving a complete set of therapeutic properties and genes associated with cannabis chemical constituents and allows for gene-property linkage to be elucidated. With knowledge of gene-property linkage, therapeutic properties can be targeted for enhancement through breeding appropriate strains, similar to how THC levels historically increased due to selective breeding for psychoactive effects (van bakel 2011). This knowledge base will enable the discovery of many such linkages, leading to focused research on acquiring more benefits from the plant. The quality of the network, however, is dependent upon the underlying NLP techniques. As a result, there can be some inaccurate relationships depicted within this network. This can be improved by incorporating a labeled data and use them to train more robust NLP models, which should improve the accuracy.

Although this network is built from existing knowledge already published in research articles, discovering more relationships is possible by mining information from this extensive network. Apart from direct links (obtained from already published articles), latent links can be established through data mining techniques on these networks, such as random walks and node embeddings. These techniques will help to identify potential relationships between genes, chemicals, and properties that are not directly observed from the data. Unearthing such potential links will boost future research by helping to generate hypotheses to prove or disprove those links empirically.

We intend to update this network periodically to incorporate information from newly published research articles into this knowledge base. Furthermore, making this network publicly available and open-sourced will enable the research community to contribute further and enrich this knowledge base at frequent intervals. These steps will help the network to grow organically and create an active community around it.

An essential aspect of future work will be the development of methods to automatically label relationships specifically, for instance the precursors and product mentioned for CBDAS. Annotating these relationships specifically will create a knowledge graph beneficial to many other research areas. In this paper, we have defined relationships based on *co-occurrences*, but in the future we will explore intra-sentence and inter-sentence relationship extraction methods to improve the coverage of relationships extracted from text. These relationship extractions methods will produce a more connected network between genes, chemicals, and therapeutic properties of *Cannabis sativa*.

The *Cannabis sativa* Genetics and Therapeutics Network serves as a starting point for downstream graph machine learning or convolutional methods that make node-level, edge-level, and graph-level predictions. These methods are currently receiving much attention (Leskovec 2021). Predicting direct and indirect relationships and predicting novel graph edges that represent new therapeutic links will be two new goals for future work.

## Conclusions

This paper introduces a natural language processing-based method to automatically create a network of *Cannabis sativa-*related genes, chemicals, and their therapeutic properties from published research articles. This network constitutes a unique cannabis-related knowledge base that replaces time-consuming and labor-intensive manual extraction of information from the massive amount of available cannabis literature from only one of the major scientific publication databases. Access to this information will benefit cannabis breeders, cannabis researchers, organic bio-chemists, pharmaceutical researchers, and many others. This publicly available network will provide the research community with a new resource, which will boost the cannabis research community and potentially open up new research questions in this emerging area by steam-lining the research process and enabling retrieval of information otherwise unavailable.

## Supplementary Information

**Additional file 1.**

## Data Availability

All datasets and code used in this research is available in the “Cannabis Genetics and Therapeutics Network” repository: https://github.iu.edu/trjojack/cannabis genetics and therapeutics network.

## Footnotes

1
https://github.iu.edu/trjojack/cannabis_genetics_and_therapeutics_network
