# Supplementary material for: The Cannabis sativa genetics and therapeutics relationship network: automatically associating cannabis-related genes to therapeutic properties through chemicals from cannabis literature
Source: J Cannabis Res. 2023 May 30;5:16. doi: 10.1186/s42238-023-00182-z (PMC10227399; doi:10.1186/s42238-023-00182-z)
Supplement: Supplementary file 1 — Additional file 1. [file 42238_2023_182_MOESM1_ESM.pdf]

Supplementary Information for

The *Cannabis sativa* Genetics and Therapeutics Network

Trever J. Jackson

Sunandan Chakraborty

Trever Jackson

Email: trjojack@alumni.iu.edu

This PDF file includes:

1. Supplementary text
2. SI References

Summary of workflow:

In order to document relationships between cannabis genetics, chemicals, and therapeutic properties we developed a network graph based on paragraph co-occurrence of entities. Initially we curate a corpus of cannabis publications and then extract the entities of interest forming a network of gene-chemical and chemical-property relationships. To document chemical profiles of cannabis strains we add a "strain" entity with an attribute that lists the documented chemical profile of the strain, including name and concentration for each chemical. We employ a dictionary look-up method with pubchempy to combat false positive chemical mentions and linking to the Gene Ontology database to combat false positive gene name mentions. Following is a detailed explanation of our web scraping and network building methods:

A1. Web scraping for the cannabis corpus and initial entity list creation

Obtaining documents from Pubmed Central required the production of a web scraping pipeline, since Pubmed Central does not provide document text data as direct output. Pubmed and Pubmed Central provide access to citation records for publications. These records include an external link for open access documents. Biopython's bio.entrez package was utilized for obtaining a list of document identifications numbers returned for search term 'Cannabis sativa'. These 30,000 IDs enabled access to full records for each document, upon which the python package beautiful soup 4 (bs4) was used to

perform “web scraping” or the “process of extracting and combining contents of interest from the Web in a systematic way”.[1] The built-in bio.entrez and bs4 functions handle requests for online information in an appropriate way as to not overload the host servers. Many non-English language documents were observed and therefore another python package, TextBlob, was employed to scan titles and omit non-English documents.

Prior to building the network, we downloaded a list of 468 cannabis chemical constituents from a study by Jagganathan et al. 2020. Pubchempy was then used to look-up each chemical in the cannabis chemical list and create a python dictionary of {cannabis chemical: all synonyms}. This dictionary facilitated building of test sets and, when building the network, directing synonyms for cannabis chemicals to a central hub node, for instance, “thc”, and “delta9-tetrahydrocannabinol” are funneled to the hub node, “tetrahydrocannabinol”. This helps to de-clutter the network and capture all relevant information in the same edge.

We also manually curated a list of 150 medicinal or therapeutic properties attributed to cannabis chemicals from several review papers. We then utilized the python package gensim to vectorize each word in the corpus. Word vectors are numerical “distributed representations of words in vector space” introduced by Mikolov et al. in 2013 [2][3]. Word vectors are often used for downstream NLP tasks. We made use of word vector similarity to locate new property entities, expanding the therapeutic property list to 755.

A2. Biological named entity recognition

We made use of three scispaCy models for chemical name and gene name entity recognition. Each model is pre-trained for bioNER of a set of biological entities. The model trained on the BIONLP13CG corpus locates many entity types including, SIMPLE_CHEMICAL and GENE OR GENE PRODUCT. The model trained on the BC5CDR corpus locates DISEASE and CHEMICAL entities, while the model trained on the CRAFT corpus locates GGP, and CHEBI entities among others, CHEBI referring to biological chemical and GGP referring to gene or gene product. The entity lists are joined by making a complete list without repeating those in common.

As discussed in the scispaCy performance section below, to combat false positive chemical name occurrences, each chemical name extracted was looked-up in the pubchempy dictionary to ensure it is truly a chemical name. Gene name occurrences were checked using scispaCy's entity linking functionality. This function allows the user to link entities to a knowledge base for purposes beyond entity recognition. We make use of linkage to the Gene Ontology knowledge base. Only gene names with linkage in Gene Ontology are kept. Cannabis chemical synonyms are routed to the name for the hub node via the chemical name synonym dictionary prior to edge building.

A3. Network formation

The structure of the html pages web scraped played a significant role in the design of our methods, particularly our relationship extraction algorithm. In html code, <p> tags denote paragraphs of text data, providing the opportunity to easily load each <p> tag from each available document into a line of a pandas data frame in python along with document ID in adjacent column. Many recent relation extraction models process data at the sentence level due to computational constraints, thereby limiting the model to within-sentence information. Zhao et al. in 2018 note that "cross-sentence relations extraction has received little attention, even in the supervised-learning setting." [5] Also noting that "causal relations naturally have an attached network structure, making it possible to leverage structural inference for causal relation discovery…" thereby putting forth a method of causal relation extraction, CausalTriad, involving establishment of triad relationships from a sliding-window of sentences; extracting entities by matching to medical databases [5]. Taking these observations into account and an additional factor, that biomedical text data can be difficult to split at the sentence level due to many unusual text elements, such as chromosome positions ("6q23.1") or references to tables or figures ("fig. 1"), we process our data at the paragraph level. We produce a network graph (fig. 2) from paragraph co-occurrence of entities to establish gene-chemical relationships and chemical-property relationships. The chemical-property relationships undergo an odds ratio comparison against a background random probability before establishing relationships.

Gene to chemical relationships are created by establishing *per paragraph* entity mention lists and either creating a new edge of weight = 1 or increasing edge weight by 1 for each pair of entities. This amounts to:

edge weight = entity 1 occurrences x entity 2 occurrences

For example, a paragraph leading to entity lists of:

[gene_A, gene_A] and [chem_B, chem_C, chem_C]

would result in two edges:

(gene_A, chem_B, weight=2 x 1= 2)

(gene_A, chem_C, weight=2 x 2= 4)

This allows more commonly reported relationships to be weighted higher, thereby quantifying the strength of each edge. Along with edge weight, each edge includes origin word for cannabis synonyms and {paragraph index: doc id} for accessing paragraph text. Including the ability to access cross-document text data backing-up the relationship in each edge provides access to robust information that is not specific to a relationship type or beholden to any model that could be trained inefficiently. This functions well for the information retrieval we desire and for the establishment of ground-level relationships from which we or others can work to refine more specific labels in the future.

For chemical to property relationships, we make use of an odds ratio technique commonly used in bioinformatics sequence analysis prior to the edge building step. Odds ratio is simply the ratio of two probabilities, (P_1/P_2). Odds ratios and log odds ratios are used in bioinformatics to compare base probability at each step in a sequence against a background probability of randomness, thereby creating position-specific scoring matrices, PSSMs, for assessing sequence similarities and to search new

sequences for known motifs. Bioinformatics DNA sequence analyses often set background probability to 0.25 for DNA analysis due to four nucleotide possibilities.

Our chemical-property edge creation method iterates through each paragraph of the corpus. For each paragraph we consider the paragraph as a single sequence, $S_{paragraph}$, of words, $[w_1 w_2 \ldots w_n]$, with length n. We then define a sequence of words for comparison, S_{random}, of the same length n, however we fill S_{random} with random single words from the corpus. For each chemical and property entity, we then establish the probability, P_1, of encountering the entity in $S_{paragraph}$ and the probability, P_2, of encountering the entity in S_{random}. P_1 is simply the number of entity occurrences, $E_{occurrences}$, divided by n.

$$P_1 = E_{occurrences} / n$$

Background probability, P_2, is derived by dividing total word slots in the paragraph, n, by the number of words in the single word vocabulary, L_{vocab}.

$$P_2 = n / L_{vocab}$$

therefore,

$$\text{Entity odds ratio} = P_1 / P_2$$

when entity odds ratio > 1, **include** entity in edge-building step,

when entity odds ratio <1, **exclude** entity from edge-building step

Entities with odds ratio greater than one are more likely to be found in $S_{paragraph}$ than in S_{random}. Two entities of differing type, each occurring with greater than random frequency, are taken to have some relation. For multi-word entities, all words must pass the threshold to be included. This method allows information from shorter paragraphs, which is likely more subject-specific and concise, to be captured by the network while guarding against long paragraphs contributing many uninformative edges to the network due to lack of subject clarity or perhaps a list or table written into the html code.

To enable investigation of cannabis strains we added to the network a strain entity with an attribute, chemical profile. The chemical profile attribute is a dictionary of key = chemical and value = concentration as percentage of dry weight. We loaded nine

cannabis strain from "Gene Networks Underlying Cannabinoid and Terpenoid Accumulation in Cannabis", Zager et al. 2019. [4]

A4. Access to cannabis network

We provide access to our project on github at:

https://github.iu.edu/trjojack/cannabis_gene_chem_prop_network

There we provide three python scripts:

1) for obtaining text data and records from Pubmed Central,

2) for extracting entities and relationships, initial build of the network, save node and edge files

3) re-build the network from saved node and edge files for use of search functions.

We also provide the necessary cannabis synonym dictionary and therapeutic property list.

In file 3 we provide many functions for exploring the network. We provide functionality to search for chemical-gene, chemical-property, gene-property relationships, as well as a function to retrieve all properties for the chemical profile of a strain. If an edge exists between two entities, all paragraphs from the cannabis corpus in which entities co-occur can be easily accessed through the appropriate function.

Assessment of ScispaCy Performance:

We made assessment of scispaCy model performance for chemical named entity recognition on two subsets of data annotated for chemical names. These subsets are:

1) all chemical names using pubchempy dictionary to annotate,

2) string matching of known cannabis constituents to annotate.

Each paragraph from the cannabis corpus was annotated accordingly for chemical name mentions and used as the ground truth set. We assess precision and recall for the pubchempy annotations dataset as the dictionary look-up method likely gives a

complete annotation, meaning false positives are actual false positives. False positives are not assessed in the cannabis constituent subset because we annotated a subset of chemicals while scispaCy searched for any chemical name. We are most interested in assessing recall for the models in order to benchmark the pre-trained language models against the more time-consuming dictionary look-up method. We are reporting new, ground level information in our network therefore low recall can be acceptable with the goal to develop better performing methods in the future. By combining all models, we achieved 80% recall on pubchempy annotated chemical names and 84% recall on cannabis constituent chemical names.

Assessment of scispaCy model performance for gene named entity recognition was performed on a set of 680 paragraphs that were annotated for gene names that are known participants of plant secondary metabolite synthesis. Recall only was assessed for gene names as precision is less important to us at the moment.

Validation of methods:

To validate that our methods can extract appropriate gene-chemical relationships, we run our web scraping and network building pipelines for two homo sapiens Y chromosome genes: SOX9 and SRY. For these two human genes, we download a list of gene-chemical relationships from the Comparative Toxicogenomics Database (CTD), a manually curated database of gene-chemical and chemical-disease relationships. Despite most cited documents in CTD not being contained in Pubmed Central, we reproduced 22% of SOX9 gene-chemical relationships and 16% of SRY gene-chemical relationships. This demonstrates our methods are capable of picking-up legitimate chemical-gene relationships and should provide quality initial data for the previously un-reported cannabis network.

SI References

1. Glez-Peña, Daniel, Anália Lourenço, Hugo López-Fernández, Miguel Reboiro-Jato, and Florentino Fdez-Riverola. "Web scraping technologies in an API world." *Briefings in bioinformatics* 15, no. 5 (2014): 788-797.

2. Mikolov, Tomas, Ilya Sutskever, Kai Chen, Greg S. Corrado, and Jeff Dean. "Distributed representations of words and phrases and their compositionality." In *Advances in neural information processing systems*, pp. 3111-3119. 2013.

3. Mikolov, Tomas, Kai Chen, Greg Corrado, and Jeffrey Dean. "Efficient estimation of word representations in vector space." *arXiv preprint arXiv:1301.3781* (2013).

4. Zager, Jordan J., Iris Lange, Narayanan Srividya, Anthony Smith, and B. Markus Lange. "Gene networks underlying cannabinoid and terpenoid accumulation in cannabis." *Plant physiology* 180, no. 4 (2019): 1877-1897.

5. Zhao, Sendong, Meng Jiang, Ming Liu, Bing Qin, and Ting Liu. "Causaltriad: toward pseudo causal relation discovery and hypotheses generation from medical text data." In Proceedings of the 2018 ACM International Conference on Bioinformatics, Computational Biology, and Health Informatics, pp. 184-193. 2018.
